# Should β-blockers be used in septic shock?

**DOI:** 10.1186/cc13878

**Published:** 2014-05-19

**Authors:** Alena Lira, Michael R Pinsky

**Affiliations:** 1 Department of Critical Care Medicine, University of Pittsburgh, 642A Scaife Hall, 3550 Terrace Street, Pittsburgh, PA 15261, USA; 2 Clinical Research, Investigation, and Systems Modeling of Acute Illness (CRISMA) Center, University of Pittsburgh, 606 Scaife Hall, 3550 Terrace Street, Pittsburgh, PA 15261, USA

## Expanded abstract

### Citation

Morelli A, Ertmer C, Westphal M, Rehberg S, Kampmeier T, Ligges S, Orecchioni A, D’Egidio A, D’Ippoliti F, Raffone C, Venditti M, Guarracino F, Girardis M, Tritapepe L, Pietropaoli P, Mebazaa A, Singer M: **Effect of heart rate control with esmolol on hemodynamic and clinical outcomes in patients with septic shock.***JAMA* 2013, **310:**1683-1691.

### Background

β-blocker therapy may control the heart rate and attenuate the deleterious effects of β-adrenergic receptor stimulation in septic shock. However, β-blockers are not traditionally used for this condition and may worsen cardiovascular decompensation related through negative inotropic and hypotensive effects.

### Methods

*Objective*: The objective of the study was to investigate the effect of the short-acting β-blocker esmolol in patients with severe septic shock.

*Design*: An open-label, randomized phase 2 study was conducted between November 2010 and July 2012.

*Setting*: The study was conducted in a university hospital ICU.

*Subjects*: Patients were in septic shock with a heart rate of 95/minute or higher requiring high-dose norepinephrine to maintain a mean arterial pressure of 65 mmHg or higher.

*Intervention*: We randomly assigned 77 patients to receive a continuous infusion of esmolol titrated to maintain a heart rate between 80/minute and 94/minute for their ICU stay and 77 patients to standard treatment.

*Outcomes*: Our primary outcome was a reduction in heart rate below the predefined threshold of 95/minute and to maintain the heart rate between 80/minute and 94/minute by esmolol treatment over a 96-hour period. Secondary outcomes included hemodynamic and organ function measures; norepinephrine dosages at 24, 48, 72, and 96 hours; and adverse events and mortality occurring within 28 days after randomization.

### Results

Targeted heart rates were achieved in all patients in the esmolol group compared with those in the control group. The median area under the curve (AUC) for the heart rate during the first 96 hours was -28/minute (interquartile range (IQR), -37 to -21) for the esmolol group versus -6/minute (95% confidence interval, -14 to 0) for the control group with a mean reduction of 18/minute (*P* < 0.001). For the stroke volume index, the median AUC for the esmolol group was 4 ml/m^2^ (IQR, -1 to 10) versus 1 ml/m^2^ for the control group (IQR, -3 to 5; *P* = 0.02), whereas the left ventricular stroke work index for the esmolol group was 3 ml/m^2^ (IQR, 0 to 8) versus 1 ml/m^2^ for the control group (IQR, -2 to 5; *P* = 0.03). For arterial lactatemia, the median AUC for the esmolol group was -0.1 mmol/l (IQR, -0.6 to 0.2) versus 0.1 mmol/l for the control group (IQR, -0.3 for 0.6; *P* = 0.007), and for norepinephrine was -0.11 μg/kg/minute (IQR, -0.46 to 0.02) for the esmolol group versus -0.01 μg/kg/minute (IQR, -0.2 to 0.44) for the control group (*P* = 0.003). Fluid requirements were reduced in the esmolol group: median AUC was 3,975 ml/24 hours (IQR, 3,663 to 4,200) versus 4,425 ml/24 hours (IQR, 4,038 to 4,775) for the control group (*P* < 0.001). We found no clinically relevant differences between groups in other cardiopulmonary variables or in rescue therapy requirements. The 28-day mortality was 49.4% in the esmolol group versus 80.5% in the control group (adjusted hazard ratio, 0.39; 95% confidence interval, 0.26 to 0.59; *P* < 0.001).

### Conclusions

For patients in septic shock, open-label use of esmolol versus standard care was associated with reductions in heart rates to achieve target levels, without increased adverse events. The observed improvement in mortality and other secondary clinical outcomes warrants further investigation.

Septic shock is a state of extreme physiologic stress associated with a wide array of hemodynamic, metabolic, and physiologic processes attempting to restore homeostasis [1]. Part of this stress response is a hyperadrenergic state associated with increased circulating catecholamines. This acute stress response is a central part of the host’s response to severe stress [2,3]. If persistent, however, this hyperadrenergic response becomes detrimental [4], manifested as stress cardiomyopathy [5], splanchnic ischemia [6], proinflammatory state [7,8], procoagulant state [9], and severe catabolism, hyperglycemia and insulin resistance [10].

Arguably, any critical illness persisting beyond a few hours may be accompanied by hyperadrenergic toxicity. Endogenous catecholamine release is upregulated 20-fold in critical illness [11], and is further compounded by exogenous catecholamines administration often required to maintain vasomotor tone [12]. Blunting this hyperadrenergic response by β-adrenergic receptor blockade may thus protect against catechol toxicity in septic shock.

Morelli and colleagues evaluated the feasibility of this hypothesis using the β_1_-blocker esmolol to reduce the heart rate (HR) in tachycardic patients with severe septic shock [13,14]. The authors’ concern regarding the effect of giving a combined negative chronotropic and inotropic agent on cardiac output and microvascular blood flow led them to perform a pilot study. In this pilot study, Morelli and colleagues prospectively observed 25 septic shock patients requiring vasopressor infusion with HR >95 beats/minute who received a titrated esmolol infusion to achieve HR <95 beats/minute. The authors observed that microvascular circulation was preserved during esmolol infusion [13].

The same authors then underwent a phase 2 clinical trial, in which patients in septic shock with HR >95 beats/minute requiring norepinephrine infusion to maintain mean arterial pressure >65 mmHg were randomized to esmolol infusion titrated to maintain the HR between 80 and 95 beats/minute or to standard therapy (77 subjects in each arm). The primary outcome was the ability to reduce the HR to the target range over 96 hours. Secondary outcomes included vasopressor and fluid requirements, hemodynamic status, organ function and 28-day adverse events and mortality. The esmolol group, compared with the control group, demonstrated significant decrease in HR (mean reduction of 18 beats/minute), decreased oxygen delivery, a slightly reduced cardiac output, comparable inotropic rescue therapy but lower norepinephrine and fluid requirement, higher stroke volume, lower arterial lactate, higher glomerular filtration rate, and lower 28-day mortality. The authors concluded that esmolol use in patients with septic shock safely achieved HR reduction to a target range without adverse events [14].

The study raises a rather provocative question: whether it is safe to use β-blocker therapy in the most critically ill patients with existing hemodynamic instability, knowing that by their negative chronotropic and inotropic effects β-blockers may worsen shock and its sequelae. This question is contrasted with the rationale that poor outcomes result from the detrimental effects of adrenergic toxicity, some of which can be counteracted by competitive antagonism at the β-receptor site [15]. Importantly, β-blockers have been shown to be safe in other critical illness [16], including trauma [17], traumatic brain injury [18], and even severe burns that are characterized by an exaggerated stress response [17].

Potentially, β-blockade may not decrease cardiac output or ventricular function if lowering the HR is offset by increased left ventricular end-diastolic volume and stroke volume [15]. The targeted lower HR was chosen arbitrarily, and it is not clear whether targeting lower or higher HR would provide a better balance between minimizing hyperadrenergic toxicity and β-adrenergic stimulation. However, the authors demonstrate that β-blocker-induced HR reductions do not increase adverse outcomes in severe sepsis. Whether other, noncardiac effects of β-blockade may lead to improved hemodynamic and organ perfusion outcomes is not known. This hypothesis seems unlikely because adrenergic modulation of vascular tone, coagulation cascade, metabolism, and immune response are mediated via α-receptors and β_2_-receptors and esmolol is a selective β_1_-blocker [7,19,20].

There are two main criticisms of this study. First is the observation that all patients required high inotropic support with levosimendan. The rationale for levosimendan is that its inotropic actions are independent of β-adrenergic receptor activation [21]. This agent is rarely used, is slow in onset and is expensive, however, and the extent to which levosimendan offsets the negative inotropic effects of esmolol, thus minimizing the detrimental impact of β-blockade on hemodynamics, cannot be determined from this study – only further randomized studies will determine this offset. Importantly, although the need for levosimendan rescue was high, this did not affect the study outcome because it was not statistically different between groups.

The second criticism is that the overall mortality was extremely high. Epidemiological data suggest that severe septic shock carries 50 to 60% mortality in the general critically ill population [22,23]. In the current study, however, the control group mortality was 80%. The authors suggest that such high mortality is accounted for by the presence of multidrug-resistant Gram-negative organisms found in their patients. Although possible, this observation remains concerning. Such high mortality could have obscured the detrimental impact of β-blockade if studied in a less sick patient cohort, because 80% mortality in the control group can hide a lot of sin.

The authors document that the use of esmolol in these patients controlled tachycardia without increasing adverse effects. Although the secondary outcomes of the esmolol group appear improved, this study was neither designed nor powered to study β-blocker benefits. The finding of lower mortality in the esmolol group was incidental and limited by the extreme control group mortality rate. However, this study does set the stage for a large clinical trial to evaluate potential benefits of esmolol use in septic patients.

## Recommendation

This is an interesting and exciting proof of principal clinical trial demonstrating that beta1-adrenergic blockade to a targeted heart rate can be administered safely in vasopressor-dependent tachycardic septic patients. Readers will find its discussion and the accompanying editorial interesting in framing clinical studies going forward.

## Abbreviations

AUC: Area under the curve; HR: Heart rate; IQR: Interquartile range.

## Competing interests

The authors declare that they have no competing interests.

## References

[B1] de Montmollin E Aboab J Mansart A Annane D Bench-to-bedside review: Beta-adrenergic modulation in sepsis Crit Care 2009 13 230 10.1186/cc8026 19863760 PMC2784350

[B2] Träger K Radermacher P Debacker D Vogt J Jakob S Ensinger H Metabolic effects of vasoactive agents Curr Opin Anaesthesiol 2001 14 157 163 10.1097/00001503-200104000-00006 17016396

[B3] Pinsky MR Is there a role for β-blockade in septic shock? JAMA 2013 310 1677 1678 10.1001/jama.2013.278478 24108438

[B4] Dünser MW Hasibeder WR Sympathetic overstimulation during critical illness: adverse effects of adrenergic stress J Intensive Care Med 2009 24 293 316 10.1177/0885066609340519 19703817

[B5] Parker MM Shelhamer JH Bacharach SL Green MV Natanson C Frederick TM Damske BA Parrillo JE Profound but reversible myocardial depression in patients with septic shock Ann Intern Med 1984 100 483 490 10.7326/0003-4819-100-4-483 6703540

[B6] Jakob SM Clinical review: splanchnic ischaemia Crit Care 2002 6 306 312 10.1186/cc1515 12225604 PMC137310

[B7] Surbatovic M Veljovic M Jevdjic J Popovic N Djordjevic D Radakovic S Immunoinflammatory response in critically ill patients: severe sepsis and/or trauma Mediators Inflamm 2013 2013 362793 24371374 10.1155/2013/362793 PMC3859159

[B8] Schmitz D Wilsenack K Lendemanns S Schedlowski M Oberbeck R β-Adrenergic blockade during systemic inflammation: impact on cellular immune functions and survival in a murine model of sepsis Resuscitation 2007 72 286 294 10.1016/j.resuscitation.2006.07.001 17118511

[B9] Amaral A Opal SM Vincent JL Coagulation in sepsis Intensive Care Med 2004 30 1032 1040 10.1007/s00134-004-2291-8 15148567

[B10] Norbury WB Jeschke MG Herndon DN Metabolism modulators in sepsis: propranolol Crit Care Med 2007 35 S616 S620 10.1097/01.CCM.0000278599.30298.80 17713418

[B11] Boldt J Menges T Kuhn D Diridis C Hempelmann G Alterations in circulating vasoactive substances in the critically ill – a comparison between survivors and non-survivors Intensive Care Med 1995 21 218 225 10.1007/BF01701475 7790607

[B12] Schmittinger CA Torgersen C Luckner G Schröder DC Lorenz I Dünser MW Adverse cardiac events during catecholamine vasopressor therapy: a prospective observational study Intensive Care Med 2012 38 950 958 10.1007/s00134-012-2531-2 22527060

[B13] Morelli A Donati A Ertmer C Rehberg S Kampmeier T Orecchioni A D'Egidio A Cecchini V Landoni G Pietropaoli P Westphal M Venditti M Mebazaa A Singer M Microvascular effects of heart rate control with esmolol in patients with septic shock: a pilot study Crit Care Med 2013 41 2162 2168 10.1097/CCM.0b013e31828a678d 23873274

[B14] Morelli A Ertmer C Westphal M Rehberg S Kampmeier T Ligges S Orecchioni A D'Egidio A D'Ippoliti F Raffone C Venditti M Guarracino F Girardis M Tritapepe L Pietropaoli P Mebazaa A Singer M Effect of heart rate control with esmolol on hemodynamic and clinical outcomes in patients with septic shock: a randomized clinical trial JAMA 2013 310 1683 1691 10.1001/jama.2013.278477 24108526

[B15] Rudiger A Singer M The heart in sepsis: from basic mechanisms to clinical management Curr Vasc Pharmacol 2013 11 187 195 23506497

[B16] Oberbeck R Kobbe P Beta-adrenergic antagonists: indications and potential immunomodulatory side effects in the critically ill Curr Med Chem 2009 16 1082 1090 10.2174/092986709787581770 19275613

[B17] Herndon DN Hart DW Wolf SE Chinkes DL Wolfe RR Reversal of catabolism by beta-blockade after severe burns N Engl J Med 2001 345 1223 1229 10.1056/NEJMoa010342 11680441

[B18] Schroeppel TJ Fischer PE Zarzaur BL Magnotti LJ Clement LP Fabian TC Croce MA Beta-adrenergic blockade and traumatic brain injury: protective? J Trauma 2010 69 776 782 10.1097/TA.0b013e3181e981b8 20938265

[B19] Hjemdahl P Larsson PT Wallén NH Effects of stress and beta-blockade on platelet function Circulation 1991 84 VI44 VI61 1683610

[B20] Träger K Radermacher P Catecholamines in the treatment of septic shock: effects beyond perfusion Crit Care Resusc 2003 5 270 276 16563117

[B21] Nieminen MS Fruhwald S Heunks LM Suominen PK Gordon AC Kivikko M Pollesello P Levosimendan: current data, clinical use and future development Heart Lung Vessel 2013 5 227 245 24364017 PMC3868185

[B22] Annane D Aegerter P Jars-Guincestre MC Guidet B CUB-Réa Network Current epidemiology of septic shock: the CUB-Réa Network Am J Respir Crit Care Med 2003 168 165 172 10.1164/rccm.2201087 12851245

[B23] Ferrer R Artigas A Levy MM Blanco J González-Díaz G Garnacho-Montero J Ibáñez J Palencia E Quintana M de la Torre-Prados MV Edusepsis Study Group Improvement in process of care and outcome after a multicenter severe sepsis educational program in Spain JAMA 2008 299 2294 2303 10.1001/jama.299.19.2294 18492971

